# Improving Adversarial Robustness of ECG Classification Based on Lipschitz Constraints and Channel Activation Suppression

**DOI:** 10.3390/s24092954

**Published:** 2024-05-06

**Authors:** Xin Chen, Yujuan Si, Zhanyuan Zhang, Wenke Yang, Jianchao Feng

**Affiliations:** 1 School of Electronic and Information Engineering (SEIE), Zhuhai College of Science and Technology, Zhuhai 519041, China; xinc21@mails.jlu.edu.cn (X.C.); zhanyuan21@mails.jlu.edu.cn (Z.Z.); yangwk21@mails.jlu.edu.cn (W.Y.); fengjc22@mails.jlu.edu.cn (J.F.); 2 College of Communication Engineering, Jilin University, Changchun 130012, China

**Keywords:** arrhythmia classification, adversarial robustness, channel-wise activation suppressing, *ℓ*_∞_ distance network

## Abstract

Deep neural networks (DNNs) are increasingly important in the medical diagnosis of electrocardiogram (ECG) signals. However, research has shown that DNNs are highly vulnerable to adversarial examples, which can be created by carefully crafted perturbations. This vulnerability can lead to potential medical accidents. This poses new challenges for the application of DNNs in the medical diagnosis of ECG signals. This paper proposes a novel network Channel Activation Suppression with Lipschitz Constraints Net (CASLCNet), which employs the Channel-wise Activation Suppressing (CAS) strategy to dynamically adjust the contribution of different channels to the class prediction and uses the 1-Lipschitz’s *ℓ*_∞_ distance network as a robust classifier to reduce the impact of adversarial perturbations on the model itself in order to increase the adversarial robustness of the model. The experimental results demonstrate that CASLCNet achieves ${ACC}_{robust}$ scores of 91.03% and 83.01% when subjected to PGD attacks on the MIT-BIH and CPSC2018 datasets, respectively, which proves that the proposed method in this paper enhances the model’s adversarial robustness while maintaining a high accuracy rate.

## 1. Introduction

Arrhythmias are a significant group of cardiovascular diseases that can cause sudden cardiac death and pose a major threat to human health [1]. The electrocardiogram (ECG) is a diagnostic tool used to non-invasively record the heart’s electrical signals. A physician typically studies and analyzes the ECG to identify the type of disease in which it was collected [2]. However, the diagnosis requires subjective judgement by doctors with extensive clinical experience, which not only consumes a large amount of healthcare resources but also does not guarantee reliability. Therefore, researchers have begun to explore the application of efficient and accurate deep neural networks (DNNs) in the field of ECG disease diagnosis and have achieved remarkable results. Oh et al. [3] proposed a novel automated system, which achieved a 98.10% accuracy in five MIT-BIH categories. Wang et al. [4] proposed an arrhythmia classification algorithm based on the multi-head self-attention mechanism (ACA-MA) and achieved a 99.4% accuracy in five categories of the MIT-BIH dataset. Kim et al. [5] adopted a residual network with a squeeze-and-excitation (SE) block and a bidirectional long short-term memory (BIL-LSTM) for arrhythmia classification and used the synthetic minority oversampling technique (SMOTE) to solve the data imbalance, and gained a 99.20%, 99.35%, and 97.05% accuracy in MITDB, AFDB, and Cinc DB, respectively. Kumar et al. [6] built a method to extract ECG features using continuous wavelet changes and used a model with SENet and lightweight context transform (LCT) for arrhythmia classification. Zeng et al. [7] proposed Fuzz-ClustNet, which use fuzzy clustering and deep learning for ECG signals detecting arrhythmia, and achieved a 98.66% and 95.79 accuracy in the MIT-BIH and PTB dataset. Recent studies have highlighted the severe threat posed by adversarial attacks to the security of DNNs, substantiated across various domains [8,9]. Adversarial examples introduce minor perturbations to the natural ECG signals, which can cause DNNs to produce erroneous results in medical diagnoses. This can potentially lead to significant medical accidents. The authors in [10] demonstrated the deceptive nature of the electrocardiogram and introduced a novel ‘cross-subject attack’. This method uses captured victim electrocardiogram short templates to map an attacker’s electrocardiogram onto the victim’s, enabling cross-device attacks with an exceptional efficacy. Chen et al. [11] conducted a study on adversarial attacks on DNN-based ECG classification systems. They proposed two attack methods based on ECG signal characteristics and introduced a smoothness metric to quantify human-perceived distances in ECG signals. In [12], generative adversarial networks were used to create fake ECG signals using victim ECG templates. Han et al. [13] proposed the Smooth Adversarial Perturbation (SAP) method, a technique specifically designed to attack ECG signal classifiers. This method applies Gaussian kernel convolution to smooth adversarial perturbations, reducing the occurrence of physiologically implausible square-wave artefacts that may arise.

The adversarial attack algorithms applied in the field of image recognition can similarly be utilized to target ECG signals. The Fast Gradient Sign Method (FGSM)**,** proposed by Goodfellow et al. [14], generates adversarial examples based on the model gradient and single-step optimization, representing a classic adversarial attack technique. Building upon the FGSM, Madry et al. [15] proposed the Projected Gradient Descent (PGD) adversarial attack algorithm. This method involves multiple iterations; a random perturbation not exceeding the specified perturbation range is superimposed on the natural example, and this is used as the initial adversarial examples for multiple iterations. The definition of PGD is provided in Equation (1):

(1) $$x_{adv}^{\prime }=Clip_{x,\epsilon }\left(x_{adv}+\delta \;*\;sign\left(\nabla_{x}J\left(x_{adv},y\right)\right)\right)$$

where x is the natural example and y is the natural example label, $x_{adv}$ denotes the initial adversarial examples and $x_{adv}^{\prime }$ is the new adversarial examples, ε is the maximum adversarial perturbation, δ is the step size of each iteration, J(⋅,⋅) calculates the predicted loss of the neural network, and $Clip_{x,\varepsilon }(\;)$ limits the size of the perturbation to the inside of the circle centred on the data x, with ε as the threshold. PGD can generate the strongest adversarial examples within the approximate sample space and stands as one of the widely used adversarial attack methods. Carlini et al. [16] proposed the C&W adversarial attack algorithm, treating adversarial examples as optimizable variables. They designed a loss function to transform the generation process of adversarial examples into a solvable optimization problem. Currently, C&W is regarded as one of the most effective white-box attack algorithms based on gradient optimization.

In order to safeguard DNNs from malicious attacks using adversarial examples on ECG signals, researchers have conducted in-depth investigations. Wiedeman et al. [17] introduced a novel ensemble method based on feature decorrelation and Fourier partitioning to enhance network features and reduce the impact of adversarial attacks. This approach aims to fortify the network against adversarial perturbations. Jeong et al. [18] proposed Defensive Adversarial Training, which involves training the model using diversified noise data to enhance the robustness of the recognition algorithm. The results demonstrate the significant effectiveness of this method in resisting noise injection and random noise compared to traditional noise removal solutions. To enhance the robustness of ECG signal classification models against adversarial noise, Ma et al. [19] introduced a regularization method based on the Noise-to-Signal Ratio (NSR). The approach aims to improve the robustness of DNNs against adversarial perturbations. Shao et al. [20] proposed a defense method based on adversarial distillation training, demonstrating its efficacy in enhancing the generalization performance of DNNs against adversarial attacks in ECG classification. The above literature does not explore the effect of the model structure on the robustness of ECG signal classification. However, recent research highlights the critical roles played by the feature extraction module and classifier in adversarial robustness [21,22,23]. In response, this research focuses on elucidating the influence of the model structure on the adversarial robustness of ECG signals. Our efforts are directed towards enhancing the model architecture, particularly the channel activation in the feature extraction stage and the design of the classifier, to improve the model’s robustness against adversarial examples in ECG signals. During the feature extraction phase, adversarial perturbations accumulate distortions in the channel activation magnitude, leading to a signal enhancement effect that renders the model prone to misclassification under adversarial attacks. Moreover, for natural examples of the same category, robust channels in the model generate more universally applicable patterns, whereas adversarial examples frequently activate non-robust channels, resulting in incorrect model outputs and diminishing network robustness [24]. In the feature extraction stage, the primary distinctions in features between adversarial and natural examples originate from variations in the channel activation magnitude and frequencies induced by adversarial perturbations, thus influencing the model’s performance in adversarial robustness. During the classification stage, adversarial attacks on the feature extraction phase induce variations in feature vectors, consequently leading to misclassifications by the classifier. Lipschitz continuity imposes a constant constraint on the range of variations between the input adversarial perturbation and the output of the model, with the minimum non-negative constant satisfying this property referred to as the Lipschitz constant for the classifier [25]. By designing a classifier with a Lipschitz constant constraint, the classification accuracy of adversarial examples can be effectively improved, particularly those with significant differences from the feature vectors of natural examples. This constraint plays a positive role in reinforcing the model’s stability and adversarial robustness.

The main contributions of this study are summarized as follows:

1. We proposed a novel robust model Channel Activation Suppression with Lipschitz Constraints Net (CASLCNet). In the feature extraction stage, CASLCNet employs the Channel-wise Activation Suppressing (CAS) strategy with an auxiliary classifier for the adaptive learning of the channel importance. This strategy dynamically adjusts channels to suppress non-robust channels. In the classification stage, CASLCNet utilizes a *ℓ*_∞_ distance network with the Lipschitz continuity as the classifier, effectively resisting small perturbations generated by adversarial attacks.
2. We employed Misclassification Aware Adversarial Training (MART), which can further improve the adversarial robustness of CASLCNet for ECG classification.
3. We validated the model adversarial robustness using multiple adversarial attack methods in the MIT-BIH dataset and the CPSC2018 dataset and compare it with state-of-the-art methods. The experimental results show that the method in this paper can effectively defend against malicious attacks on the model by multiple adversarial attack methods while maintaining a high accuracy, and outperforms the state-of-the-art methods in a variety of metrics.

## 2. Materials

### 2.1. Datasets

This study validates the performance of the CASLCNet network using the processed MIT-BIH dataset [26,27] and the CPSC2018 dataset [28]. The specific details of the dataset are presented in Table 1.

1. The MIT-BIH dataset, sampled at a frequency of 125 Hz, is categorized into five classes based on AAMI standards, resulting in a total of 109,446 samples.
2. The CPSC2018 dataset, sampled at a frequency of 500 Hz with a duration ranging from 6 to 60 s, comprises nine sample classes. To streamline our experimentation, 477 electrocardiogram samples with multiple labels are excluded, leaving 6400 samples of 12-lead electrocardiograms for further analysis.

### 2.2. Validation Metrics

This study employs two fundamental evaluation metrics: accuracy and F1 score. Additionally, $ACC_{robust}$ and $F1_{robust}$ [19], as two evaluation metrics, are utilized to measure the overall robustness of the network within a specific attack range. The definition of $ACC_{robust}$ is provided in Equation (2):

(2) $$ACC_{robust}=\sqrt{ACC_{clean}\times AUC_{∥\epsilon {∥}_{\infty }\leq \epsilon_{max}}}$$

Additionally,$ACC_{clean}$ represents the normalized classification accuracy, and $AUC_{||\epsilon |{|}_{\infty }\leq \epsilon_{max}}$ is the normalized area under the curve in the presence of varying noise levels. It calculates the average classification accuracy within a certain range of noise levels $||\epsilon |{|}_{\infty }\leq \epsilon_{max}$. Therefore, $ACC_{robust}$ is able to provide a comprehensive performance indicator that measures both a clean sample accuracy and overall robustness. Similarly, the definition of another metric $F1_{robust}$ is given by Equation (3):

(3) $$F1_{robust}=\sqrt{F1_{clean}\times AUC_{∥\epsilon {∥}_{\infty }\leq \epsilon_{max}}}$$

Due to the potential significant impact of small adversarial perturbations on the model output, a maximum perturbation $\epsilon_{max}$ is set to consider only a specific range of perturbation $||\epsilon |{|}_{\infty }\leq \epsilon_{max}$. In this experiment, for the MIT-BIH dataset with $\epsilon_{max}\;$of 0.3, the ranges of perturbation are set to $\left\{0.01,0.03,0.05,0.1,0.2,0.3\right\}$ and the number of iterations is set to 100. In the CPSC2018 dataset, $\epsilon_{max}\;$is 0.1 and the ranges of perturbation are set to $\left\{0.001,0.003,0.005,0.007,0.01,0.03,0.05,0.1\right\}$ and the number of iterations is set to 100. In the SAP attack algorithm, gaussian kernel with size s is set to$\;\left\{5,7,11,15,19\right\}$, and standard deviation σ is set to $\left\{1.0,3.0,5.0,7.0,10.0\right\}$.

## 3. Methods

**Figure 1:**
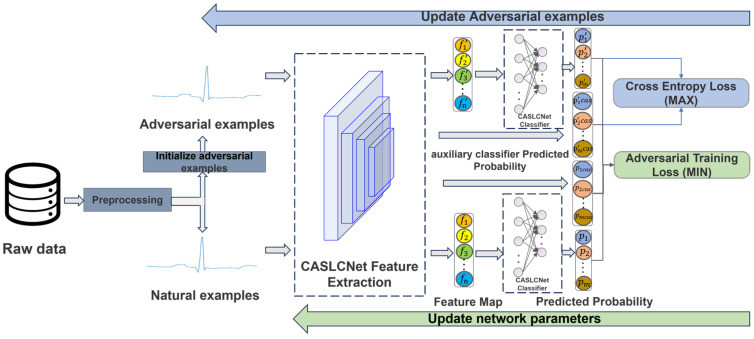
Overall flow of the proposed methodology.

In this section, the adversarial robustness model for arrhythmia classification using CASLCNet is described in detail, and the overall flowchart is shown in Figure 1. Firstly, the ECG signals in the dataset are preprocessed and initialized to generate adversarial examples; the natural examples and the adversarial examples are fed into CASLCNet for feature extraction to obtain the feature vectors and the auxiliary classifier prediction probabilities, respectively, and the feature vectors are fed into the *ℓ*_∞_ distance network as the classifier to obtain the prediction probabilities. The above data are valued for adversarial training, i.e., maximizing the Cross-Entropy loss function to find the worst-case samples, minimizing the adversarial loss function to train a model that is robust to the adversarial examples, and constantly updating the model network parameters while updating the adversarial examples for training, in the hope of obtaining a model with better adversarial robustness.

### 3.1. Data Preprocessing

Preprocessing of ECG data is required before model training. The MIT-BIH dataset needs to perform up-sampling operations on the training and test sets to eliminate the effect of class imbalance on model training. The CPSC2018 dataset needs to populate the ECG data less than 60 S with 0 to 60 S at both ends, and the ECG data more than 60 S with the first 60 S intercepted. Since leads V3, V4, V5, and V6 can be obtained from other leads, these four leads are removed and the remaining eight leads are saved. Using feature normalization, the lead values are deflated to between −1 and 1 according to the maximum value of each lead, again using up-sampling to eliminate the effect of category imbalance.

### 3.2. Feature Extraction and Classification (CASLCNet)

**Figure 2:**
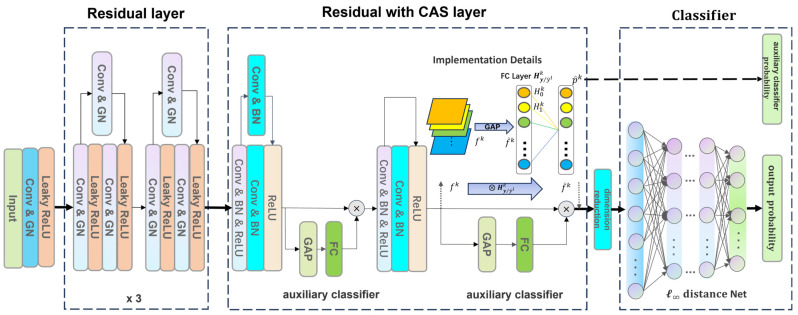
CASLCNet framework.

**Figure 3:**
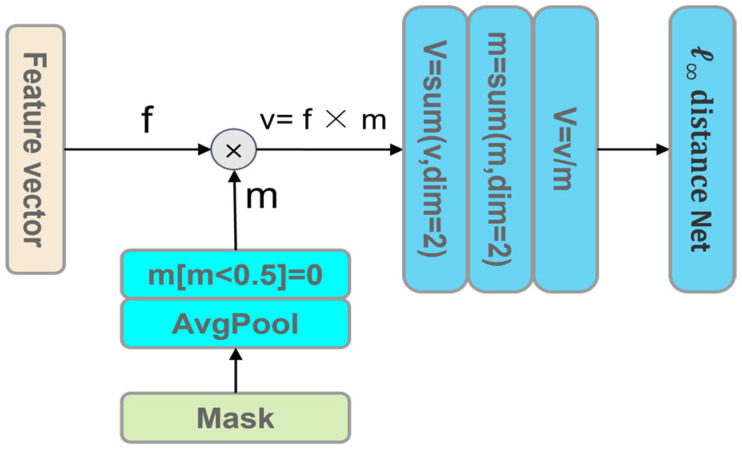
Dimension reduction diagram.

In this section, CASLCNet is described in detail, and the general framework is shown in Figure 2. The network consists of residual modules [29], residual modules with Channel-wise Activation Suppression strategy [30], and *ℓ*_∞_ distance network [31]. In the feature extraction stage, the residual module and the residual module with Channel-wise Activation Suppression strategy are used to extract the deep features of the ECG signal while dynamically adjusting the channel importance. Meanwhile, using the *ℓ*_∞_ distance network as the classifier can constrain the Lipschitz constant of the classifier, which can effectively resist the influence of adversarial perturbations on the model output. The details of the model will be introduced one by one in the following. Since the data in the CPSC2018 dataset are variable-length data, zero padding is used in the preprocessing stage to fix the length of the ECG signal to make it easier to feed into the model for computation. To eliminate the effect of zero padding in the lead data on the model, average pooling is performed using a fixed-length input mask in the dimensionality reduction stage in CASLCNet and multiplied with the extracted feature vectors to obtain a valid output vector. After the output masking operation, channel averaging weighted by the mask is performed to reduce the dimensionality of the output vectors from variable-length to a fixed length, which is then fed into the *ℓ*_∞_ distance network for classification. The specific flowchart is shown in Figure 3. Dimension reduction is carried out using global average pooling and dimension transformation in the MIT-BIH dataset.

#### 3.2.1. Residual Module

The expressive capability of DNNs gradually increases with the addition of layers, allowing for higher accuracy and stronger performance through deeper network architectures. However, the augmentation of depth in neural networks poses challenges such as overfitting, gradient explosion, gradient vanishing, and a decline in information propagation capability, leading to a degradation in model performance. In addressing these challenges, He et al. [29] introduced the concept of residual learning into deep neural networks and proposed residual network ResNet.

The residual module used in this paper is modified from the original residual module, consisting of convolutional layers, group normalization layers, activation functions, and a skip connection, is designed to learn deeper-level features. This module adds the input of the module to the output obtained after convolutional operations. This mechanism facilitates the transfer of gradient information between layers, overcoming issues like gradient vanishing. Consequently, the model’s overall performance is enhanced.

#### 3.2.2. Channel-Wise Activation Suppression Strategy

During the model training phase, the Channel-wise Activation Suppression strategy dynamically learns the importance of channels. By suppressing non-robust channels, it effectively reduces the activation magnitude of feature vector channels and the frequency of network channel activations. Let the k-th CAS module receive the feature vector activated by the residual module through the ReLU function $f^{k}\in R^{W\times C}$, where C represents the number of channels in the feature vector, and W is the width of the feature vector. Initially, the feature vector $f^{k}$ is subjected to global average pooling to obtain the channel activation vector ${\hat{f}}^{k}$. Subsequently, this vector ${\hat{f}}^{k}$ is fed into a fully connected layer for category classification. Assuming there are M classes in the dataset, the parameters of the fully connected layer for the auxiliary classifier can be expressed as follows: $H^{k}=[H_{1}^{k},H_{2}^{k},..,H_{C}^{k}]\in R^{C\times M}$. The fully connected layer identifies the importance of each channel for a given class, and reweights the natural feature vector accordingly, followed by forward propagation to the next layer.

During the model training phase, the heartbeat data label y is used as an indicator for determining the importance of classes, denoted as $H_{y}^{k}\in R^{c}$. As obtaining data label information is not possible during the testing phase, the predicted class by the auxiliary classifier is used as channel weights, denoted as $H_{{\hat{y}}^{k}}^{k}\in R^{C}$. For adversarial examples $x_{adv}$, the output probability of the auxiliary classifier is ${\hat{p}}^{k}=softmax({\tilde{f}}^{k}H^{k})\in R^{M}$. Let K represent the total number of CAS modules in the network, and the loss function used for network training is L. The overall loss function for CAS modules during network training is defined in Equation (4):

(4) $$L_{CAS}\left({x,x}_{adv},y;\theta ,H\right)=\frac{\alpha }{K}\cdot \sum_{k=1}^{K}\;L\left({\hat{p}}^{k}\left({x,x}_{adv},\theta ,H\right),y\right)$$

Among other things, θ denotes the model parameters, and α is the hyperparameter that balances the training of CAS modules.

##### 3.2.3. *ℓ*_∞_ Distance Network

The *ℓ*_∞_ distance network is a *ℓ*_∞_ multilayer perceptron network composed of distance neurons as fundamental units. *ℓ*_∞_ distance neurons take the feature vector $x_{f}$ as input, with an added bias term b. The *ℓ*_∞_ parametric distance can be computed by the norm distance between $x_{f}$ and the parameter w, as defined by Equation (5):

(5) $$u\left(x_{f},\left\{w,b\right\}\right)=∥x_{f}-w{∥}_{\infty }+b$$

Based on the definition of *ℓ*_∞_ distance neurons, a fully connected *ℓ*_∞_ distance network can be constructed. The *ℓ*_∞_ distance network module g takes ${x_{f}}^{\left(0\right)}=x_{f}$ as input, and its L-th layer, ${x_{f}}^{\left(l\right)}$, is defined as Equation (6):

(6) $$x_{fi}^{\left(l\right)}=u\left({x_{f}}^{\left(l-1\right)},\left\{w^{\left(l,j\right)},b_{i}^{\left(l\right)}\right\}\right)=∥{x_{f}}^{\left(l-1\right)}-w^{\left(l,i\right)}{∥}_{\infty }+b_{i}^{\left(l\right)},l\in \left[L\right],i\in \left[n_{l}\right]$$

Here, $n_{l}$ represents the number of neurons in the l-th layer. For a classification problem with M classes, $n_{L}=M$. The $l_{\infty }$ distance network module takes $g(x_{f})={x_{f}}^{\left(L\right)}$ as output probabilities and predicts the class $arg\underset{i\in [M]}{max}\;[g(x_{f}){]}_{i}$. Due to the 1-Lipschitz mapping property of the distance layer concerning norms, any *ℓ*_∞_ distance network is inherently 1-Lipschitz through composition.

### 3.3. Misclassification-Aware Adversarial Training

In this study, we employed Misclassification Aware Adversarial Training [32] to train the network. For an M-class classification problem, we are given a dataset $\{(x_{i},y_{i}){\}}_{i=1,\ldots ,n}$, where the natural example $x_{i}\in R^{d}$ and $y_{i}\in \{1,\ldots ,M\}$ represent the class. For a deep neural model $h_{\theta }$ with network parameters θ, an adversarial sample $x_{adv}$ is generated based on the natural example $x_{i}$. During adversarial training, both the natural examples and adversarial examples are fed into the $h_{\theta }$ to obtain model prediction probability values. Samples with model prediction errors are classified into three categories: natural example prediction error $h_{\theta }(x_{i})\neq y_{i}$, adversarial sample prediction error $h_{\theta }(x_{adv})\neq y_{i}$, and inconsistency in predictions between the natural examples and adversarial examples $h_{\theta }(x_{i})\neq h_{\theta }(x_{adv})$. MART addresses these three types of misclassifications during adversarial training, and its loss function is defined by Equation (7):

(7) $$L^{\mathrm{MART}}\left(x,x_{adv},y;\theta \right)=\lambda \cdot KL\left(p\left(x_{i},\theta \right)∥p\left(x_{adv},\theta \right)\right)\cdot \left(1-p_{y_{i}}\left(x_{i},\theta \right)\right)\;\;+\frac{1}{n}\cdot \sum_{i=1}^{n}\;BCE\left(p\left(x_{adv},\theta \right),y_{i}\right)$$

Here, boosted cross-entropy (BCE) loss is used for misclassifications where $h_{\theta }(x_{adv})\neq y_{i}$, allowing the model to obtain a stronger decision boundary. For misclassifications where $h_{\theta }(x_{i})\neq h_{\theta }(x_{adv})$, KL divergence is employed to minimize the distribution difference between the two, thereby better fitting the outputs of the natural examples and adversarial examples. Regarding misclassifications where $h_{\theta }(x_{i})\neq y_{i}$, the soft decision $\left(1-p_{y_{i}}(x_{i},\theta )\right)$ dynamically adjusts the loss function size for improved robust network training. In the training of the model in this study, MART is also applied to the auxiliary classifier. Therefore, the overall loss function during network training is given by Equation (8):

(8) $$L=L^{\mathrm{MART}}\left(x,x_{adv},y;\theta \right)+\frac{\alpha }{R}\cdot \sum_{r=1}^{R}\;L_{CAS}^{\mathrm{MART}}\left(x,x_{adv},y;\theta ,H\right)$$

The direct use of MART for the CPSC2018 dataset leads to difficulties in model convergence. Therefore, it is necessary to add the MSE loss function to assist the model convergence during the training process. The overall loss function formula is given by Equation (9):

(9) $$L=L^{\mathrm{MART}}\left(x,x_{adv},y;\theta \right)+\frac{\alpha }{R}\cdot \sum_{r=1}^{R}\;L_{CAS}^{\mathrm{MART}}\left(x,x_{adv},y;\theta ,H\right)+\frac{1}{n}\cdot \sum_{i=1}^{n}\;{\left({y_{i}-h}_{\theta }\left(x_{i}\right)\right)}^{2}$$

## 4. Result and Discussion

### 4.1. Experimental Setup

The research experiments are conducted on a server equipped with an Intel(R) Xeon(R) Gold 5218 CPU (2.30 GHz) and NVIDIA A100-SXM4 GPU (40 GB memory). The operating system used is Centos 8, with Python version 3.8.3, PyTorch version 1.13.1, and CUDA version 11.6. For the MIT-BIH dataset, the batch size is set to 512, the number of training rounds is 100, the Adamax optimizer is used for training, the initial learning rate is set to 1 × 10^−3^, and the ReduceLROnPlateau learning rate scheduler is used to dynamically adjust the learning rate. For CASLCNet training, the PGD attack algorithm is used to generate adversarial examples for adversarial training, the number of attacks is set to 10, the attack range is 0.1, and the attack step size is one-tenth of the attack range. For the CPSC2018 dataset, the batch size during training is set to 64, the initial learning rate is set to 1 × 10^−4^, and the model is also trained using MART with the number of attacks set to 10, the attack range to 0.01, and the rest of the settings the same as for the MIT-BIH dataset. Table 2 shows the detailed structure of the network using CASLCNet for the MIT-BIH and CPSC2018 dataset.

### 4.2. Channel-Wise Activation Suppression Effect

**Figure 4:**
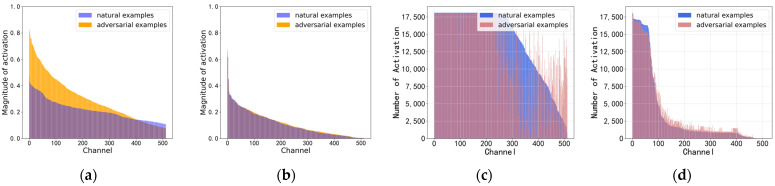
Illustration of channel-wise activation frequency and magnitude using CASLCNet and Resnet18 on MIT-BIH datasets. (**a**) Channel-wise activation magnitude of Resnet18; (**b**) channel-wise activation magnitude of CASLCNet; (**c**) channel-wise activation frequency of Resnet18; and (**d**) channel-wise activation frequency of CASLCNet.

**Figure 5:**
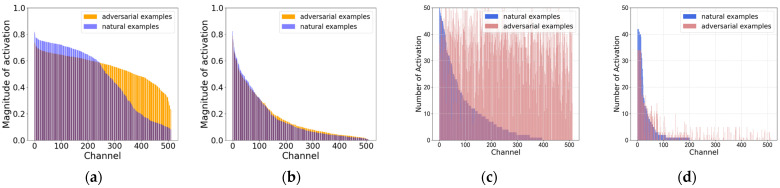
Illustration of channel-wise activation frequency and magnitude using CASLCNet and Resnet18 on CPSC2018 datasets. (**a**) Channel-wise activation magnitude of Resnet18; (**b**) channel-wise activation magnitude of CASLCNet; (**c**) channel-wise activation frequency of Resnet18; and (**d**) channel-wise activation frequency of CASLCNet.

As examples, we chose the N class and Normal class from the MIT-BIH and CPSC2018 dataset. Our observation focused on the channel activation frequency and magnitude at the final layer of the model’s feature extraction. For each channel, if the activation value surpassed a threshold (20% of the maximum activation value across all 512 channels in MIT-BIH, and 70% of the maximum activation value in CPSC2018), the channel is identified as an activated channel. Subsequently, we calculated the activation frequency on each channel for both the natural examples and adversarial examples, sorting them in descending order of the natural example’s activation frequency. In the experiments, CASLCNet is trained using MART and ResNet18 is trained using the cross-entropy loss function as the contrast model. Figure 4 and Figure 5 illustrates the channel-wise activation frequency and magnitude of ResNet18 and CASLCNet on the test sets of both datasets. From the subfigures a, it is evident that the channel-wise activation magnitude of the adversarial examples is significantly higher than that of the natural examples. This indicates that adversarial perturbations progressively accumulate from the model’s input layer to the output layer. By looking at the subfigures c, we notice that adversarial examples activate the model channels more uniformly, frequently activating non-robust channels seldom activated by the natural examples. This has a severe impact on the model’s robustness. Subfigures b depict the activation magnitude of CASLCNet when faced with adversarial examples. It is apparent that our proposed method effectively suppresses the activation magnitude of adversarial examples, reducing the magnitude gap between adversarial and natural examples. Subfigures d represent the channel-wise activation frequency of adversarial examples. Our proposed method effectively suppresses the channel activation frequency, aligning the activation frequencies of natural examples and adversarial examples and reducing the activation on non-robust channels by adversarial examples. Consequently, this mitigates the impact of adversarial sample attacks on the network, enhancing overall robustness.

### 4.3. Hyperparameter Selection Experiment

**Figure 6:**
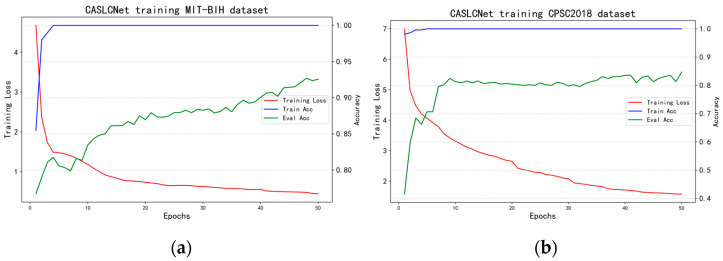
CASLCNet network loss function and accuracy display in MIT-BIH and CPSC2018 dataset. (**a**) CASLCNet indicator in MIT-BIH dataset; and (**b**) CASLCNet indicator in CPSC2018 dataset.

In the training of the CASLCNet network, adjustments to the α parameter of the CAS loss are made to achieve optimal training outcomes. To assess the sensitivity of the CAS strategy under different α values, MART is conducted on the MIT-BIH and CPSC2018 datasets for α values of $\left\{0,1,2,3,4\right\}$, where α=0 represents standard adversarial training. Table 3 presents the corresponding ${ACC}_{robust}$ and ${\;F1}_{robust}$ scores for each α value. The results indicate that the model achieves optimal performance across metrics when α is set to 2, striking a balance between the accuracy rate and robustness. Figure 6 shows the loss function curves as well as the accuracy curves of CASLCNet in the MIT-BIH dataset and the CPSC2018 dataset when the hyperparameter α is 2.

### 4.4. Adversarial Robustness Verification

To assess the effectiveness of CASLCNet in defending against various malicious attacks, we conducted validation using different adversarial attack methods on the test sets of the MIT-BIH and CPSC2018 datasets. The adversarial attack methods employed included white noise, FGSM, C&W, PGD, and SAP. White noise and FGSM attacks utilized a single iteration, while MI-FGSM and C&W used 100 iterations. White noise and FGSM and MI-FGSM perturbation ranges are set to 0.1 and 0.01 in the MIT-BIH and CPSC2018 datasets, respectively, with an MI-FGSM step range of 0.01 and 0.001. When using PGD and SAP adversarial attacks, the settings are as shown in 2.2. Table 4 provides the detailed accuracy and F1 scores under the white noise, FGSM, MI-FGSM, and C&W attack methods. Table 5 and Table 6 show the accuracy and F1 scores of CASLCNet when the MIT-BIH dataset and the CPSC2018 dataset are attacked by different PGD and SAP adversarial attack, respectively. Notably, the model’s accuracy and F1 scores showed minimal degradation when faced with white noise, FGSM, MI-FGSM, C&W, and SAP attacks. Even under PGD adversarial attacks, the model maintained a high level of accuracy, demonstrating the model’s ability to effectively withstand various adversarial attacks while preserving a high accuracy.

### 4.5. Ablation Experiment

To assess the effectiveness of each module in enhancing the adversarial robustness of the CASLCNet network, we conducted ablation experiments on both the MIT-BIH and CPSC2018 datasets. Method 1 employed ResNet18 as the baseline model, while Method 2 replaced the last feature extraction layers of Method 1 with residual modules incorporating the channel-wise activation suppression strategy. Method 3 replaced the fully connected layer of Method 1 with an *ℓ*_∞_ distance network serving as the classifier. Method 4 represents our proposed CASLCNet. All methods utilized MART. Table 7 shows the ${ACC}_{robust}$ and ${F1}_{robust}$ scores under the test set of MIT-BIH and CPSC2018 datasets, where × means that the method model does not contain the module, and √ means that the model contains the module, and it can be observed that Method 4 achieves the best values in all the metrics; it shows that both modules added in this paper are effective in improving the model adversarial robustness.

### 4.6. Contrast Experiment

In this study, CASLCNet is trained using various methodologies, including standard adversarial training [15], TRADES adversarial training [33], and MART. The experimental results are presented in Table 8. Table 8 demonstrates that Misclassification-Aware Adversarial Training consistently achieves optimal values across metrics. To verify the effectiveness of the CASLCNet model, three classical networks, VGG19 [34], ResNet18 and DenseNet [35], are used in this paper and the proposed CASLCNet is trained with different loss functions, respectively, and the experimental results are shown in Table 9 and Table 10, respectively. Table 9 shows the detailed results of the ${ACC}_{robust}$ and ${F1}_{robust}$ scores of each method in the MIT-BIH dataset, and Table 10 shows the detailed results of the ${ACC}_{robust}$ and ${F1}_{robust}$ scores of each method in the CPSC2018 dataset. Table 10 shows that the ${ACC}_{robust}$ and ${F1}_{robust}$ scores of CASLCNet are higher when the model is attacked by adversarial examples when trained with the cross-entropy loss function, compared to the ${ACC}_{robust}$ and ${F1}_{robust}$ scores of VGG19, ResNet18, and DenseNet trained with MART, which effectively shows that the CASLCNet network proposed in this paper has strong adversarial robustness without adversarial training. The ${ACC}_{robust}$ and ${F1}_{robust}$ scores of CASLCNet under PGD adversarial attack can be improved by more than 40% compared to other methods when CASLCNet is trained using MART in the CPSC2018 dataset. Table 9 and Table 10 show that the proposed method can achieve the best performance in each index, and can effectively improve the ${ACC}_{robust}$ and ${F1}_{robust}$ scores compared with the other networks, which indicates that CASLCNet can achieve the advantage in the accuracy of the natural examples, as well as the robustness. This observation substantiates the effectiveness of CASLCNet in significantly enhancing the model’s adversarial robustness.

### 4.7. Comparison with Existing Literature

**Figure 7:**
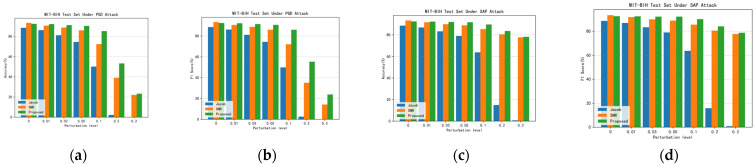
Accuracy and F1 scores of proposed method with Jacob and SNR under different adversarial attacks in MIT-BIH dataset. (**a**) Accuracy under different adversarial perturbations in PGD adversarial attacks; (**b**) F1 score under different adversarial perturbations in PGD adversarial attacks; (**c**) accuracy under different adversarial perturbations in SAP adversarial attacks; and (**d**) F1 score under different adversarial perturbations in SAP adversarial attacks.

**Figure 8:**
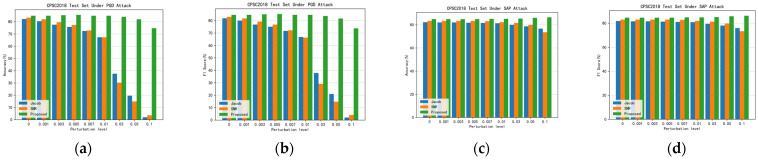
Accuracy and F1 scores of proposed method with Jacob and SNR under different adversarial attacks in CPSC2018 dataset. (**a**) Accuracy under different adversarial perturbations in PGD adversarial attacks; (**b**) F1 score under different adversarial perturbations in PGD adversarial attacks; (**c**) accuracy under different adversarial perturbations in SAP adversarial attacks; and (**d**) F1 score under different adversarial perturbations in SAP adversarial attacks.

Recent studies have indicated that SNR regularization enhances network robustness by suppressing the Signal-to-Noise Ratio (SNR) of adversarial noise signals, while Jacobian regularization mitigates the impact of adversarial noise perturbations by penalizing large gradients relative to the output. These regularization methods represent advanced approaches for defending against adversarial attacks on electrocardiographic signals. Figure 7 and Figure 8 show the histograms of the accuracy and F1 scores of the proposed method with the two methods, Jacob, as well as SNR, under different PGD and SAP adversarial attacks. Table 11 shows the detailed data of the comparison between the proposed method and the existing literature, from which it can be seen that the proposed method in this paper achieves the optimal results in terms of ${ACC}_{robust}$ and ${F1}_{robust}$ scores. In the CPSC2018 dataset, when attacked by PGD, the ${ACC}_{robust}$ and ${F1}_{robust}$ scores of this paper’s method are more than 30% higher than other methods. In the MIT-BIH dataset, this paper’s method is also reaching the best index. The above experiments fully prove that the proposed method in this paper achieves a better balance between identifying natural examples and adversarial examples; not only can it maintain a high accuracy, but it can also effectively resist the attack of adversarial examples, which effectively indicates that the proposed method in this paper is better than the existing methods in the literature and reaches the advanced level.

This study aims to investigate the effectiveness of the proposed CASLCNet in defending against adversarial examples when applied to medical diagnostics using electrocardiographic signals. We conducted experiments focusing on Channel-wise Activation Suppression, hyperparameter selection, robustness validation, and comparisons with existing literature.

In our observations, CASLCNet demonstrated significant advantages when confronted with various adversarial attack methods on the test sets of the MIT-BIH and CPSC2018 datasets. Under white noise, FGSM, MI-FGSM, C&W, PGD, and SAP attacks, CASLCNet maintained a high accuracy and F1 scores, showcasing its robust resistance to diverse adversarial attack methods. Furthermore, we conducted an in-depth investigation into the efficacy of the Channel-wise Activation Suppression strategy within CASLCNet. By scrutinizing the channel activation frequencies and magnitude of CASLCNet in the MIT-BIH and CPSC2018 datasets, we observed a significant reduction in the activation magnitude of adversarial examples. This reduction resulted in a diminished Magnitude gap between adversarial and natural examples, indicating a pivotal role played by the Channel-wise Activation Suppression strategy in effectively enhancing the model’s robustness. In terms of model training, we used an adversarial training approach that emphasizes misclassification. This approach involves designing the loss function to address misclassifications of both natural examples and adversarial examples. The results demonstrated that CASLCNet consistently achieved a favorable performance on the MIT-BIH and CPSC2018 datasets. These experiments serve as empirical evidence of the effectiveness of proposed method in enhancing the model’s robustness. Comparisons with the existing literature demonstrated that CASLCNet consistently achieved the best results in terms of ${ACC}_{robust}$ and ${F1}_{robust}$ scores, showcasing its significant advantage in adversarial attacks. This establishes CASLCNet as an advanced technology in the field of robustness research.

The proposed method in this paper achieves a significant robustness improvement in the context of arrhythmia classification, which can be applied to the automatic diagnosis of arrhythmia models, and can effectively prevent attackers from causing misdiagnosis leading to medical accidents by formulating specific adversarial examples to deceive the model, which is of great significance for improving the reliability and safety of ECG signal processing systems in practical medical applications. However, there are still some problems that need to be improved in the method of this paper. For multi-lead ECG signals, this paper’s method does not consider the influence of each lead’s signals on the robustness of the model, and there is the problem of the long computation time and large computation volume. In future work, we will investigate other applications in the field of ECG signal classification, such as identity recognition, and consider methods such as introducing a lead attention mechanism to further investigate the effect of each lead on the adversarial robustness of the model, as well as lightening the modules in CASLCNet by optimizing the adversarial training algorithms to reduce the time and computational volume.

## 5. Conclusions

In order to enhance the robustness of the electrocardiographic signal classification model, this paper proposes a novel robust network, CASLCNet. By leveraging a Channel-wise Activation Suppression strategy, CASLCNet dynamically adjusts the importance of channels, reducing the activation frequency of non-robust channels. Simultaneously, the introduction of an *ℓ*_∞_ distance network composed of *ℓ*_∞_ distance neurons serve as the network classifier, effectively suppressing the impact of adversarial perturbations on the network. This design allows the network to maintain a high accuracy while robustly resisting malicious attacks on adversarial examples. The experimental results demonstrate that our proposed method outperforms the existing literature, reaching optimal levels on the MIT-BIH and CPSC2018 datasets. This substantiates the effectiveness of our approach in defending against adversarial attacks.

## Figures and Tables

**Table 1:** Dataset details.

Dataset	Sampling Rates (Hz)	ECG Type	Number of Heartbeats	
			Training Set	Test Set
MIT-BIH	125	N	72,471	18,118
		S	2223	556
		V	5788	1448
		F	641	162
		Q	6431	1608
CPSC2018	500	Normal	868	50
		AF	926	50
		I-AVB	636	50
		LBBB	129	50
		RBBB	1483	50
		PAC	482	50
		PVC	557	50
		STD	734	50
		STE	135	50

**Table 2:** Detailed settings of CASLCNet.

Layer Name	MIT-BIH		CPSC2018	
	Output Size	Module Parameters (Kernel Size ks, Dimension d, Stride st)	Output Size	Module Parameters (Kernel Size ks, Dimension d, Stride st)
1D convolution layer	64 × 94	ks=11, st=2, d = 64	64 × 16,896	ks=11, st=2, d = 64
Group Normalization	64 × 94	[64,64]	64 × 16,896	[64,64]
Leaky ReLU	64 × 94	-	64 × 16,896	-
Residual layer 1	128 × 24	$\left[\begin{array}{c} ks=3,d=128\\ ks=3,d=128 \end{array}\right]\times 2,\;st$ = 2	128 × 4224	$\left[\begin{array}{c} ks=3,d=128\\ ks=3,d=128 \end{array}\right]\times 2,\;st$ = 2
Residual layer 2	256 × 6	$\left[\begin{array}{c} ks=3,d=256\\ ks=3,d=256 \end{array}\right]\times 2,\;st$ = 2	256 × 1056	$\left[\begin{array}{c} ks=3,d=256\\ ks=3,d=256 \end{array}\right]\times 2,\;st$ = 2
Residual layer 3	512 × 2	$\left[\begin{array}{c} ks=3,d=512\\ ks=3,d=512 \end{array}\right]\times 2,\;st$ = 2	512 × 264	$\left[\begin{array}{c} ks=3,d=512\\ ks=3,d=512 \end{array}\right]\times 2,\;st$ = 2
Residual with CAS layer	512 × 2	$\left[\begin{array}{c} ks=3,d=512\\ ks=3,d=512 \end{array}\right]\times 2,\;st$ = 1	512 × 132	$\left[\begin{array}{c} ks=3,d=512\\ ks=3,d=512 \end{array}\right]\times 2,\;st$ = 2
Downscaling (average pooling)	512	st = 2	512	ks=3072, st = 234
*ℓ*_∞_ distance network	5	$\left\{512,512,512,512,512,5\right\}$	9	$\left\{512,512,512,512,512,5\right\}$

**Table 3:** Impact of hyperparameter α on model adversarial robustness (%).

Dataset	Hyperparameter α	PGD		SAP	
		${ACC}_{robust}$	${F1}_{robust}$	${ACC}_{robust}$	${F1}_{robust}$
MIT-BIH	0	60.68	56.63	63.12	59.37
	1	87.33	87.48	88.15	88.54
	2	**91.03**	**91.22**	**91.90**	**92.07**
	3	85.50	85.72	86.09	86.33
	4	86.77	86.93	87.21	87.45
CPSC2018	0	80.61	80.46	81.84	81.70
	1	79.18	78.85	83.52	83.27
	2	**83.01**	**82.64**	**85.34**	**85.07**
	3	80.01	79.99	81.62	81.66
	4	78.78	78.32	80.75	80.35

**Table 4:** Performance metrics of CASCLC-Net in MIT-BIH and CPSC2018 dataset under different adversarial attack scenarios (%).

Dataset	Metrics	-	White Noise	FGSM	MI-FGSM	C&W
MIT-BIH	Accuracy	92.44	91.58	89.56	88.43	86.25
	F1	92.48	92.09	90.34	88.49	86.72
CPSC2018	Accuracy	84.89	84.89	86.67	61.33	60.89
	F1	84.61	85.07	86.46	62.34	57.38

**Table 5:** Performance metrics of CASCLC-Net in MIT-BIH dataset under PGD and SAP adversarial attack scenarios (%).

Attack	Metrics	0	0.01	0.03	0.05	0.1	0.2	0.3
PGD	Accuracy	92.44	92.18	91.19	90.39	85.26	53.28	23.24
	F1	92.48	92.24	91.37	90.70	85.97	55.30	23.82
SAP	Accuracy	92.44	92.30	91.96	91.69	89.63	83.58	78.17
	F1	92.48	92.38	92.13	92.12	90.04	84.06	78.71

**Table 6:** Performance metrics of CASCLC-Net in CPSC2018 dataset under PGD and SAP adversarial attack scenarios (%).

Attack	Metrics	0	0.001	0.003	0.005	0.007	0.01	0.03	0.05	0.1
PGD	Accuracy	84.89	84.89	85.33	85.56	84.89	84.89	84.00	82.00	74.67
	F1	84.61	84.61	85.07	85.30	84.63	84.63	83.75	81.68	73.76
SAP	Accuracy	84.89	84.89	84.89	84.89	85.11	85.11	85.33	86.00	86.44
	F1	84.61	84.61	84.61	84.61	84.85	84.85	85.09	85.77	86.20

**Table 7:** Results of ablation experiments (%).

Dataset	Method	BackBone	CAS Block	*ℓ*_∞_ Distance Net	PGD		SAP	
					${ACC}_{robust}$	${F1}_{robust}$	${ACC}_{robust}$	${F1}_{robust}$
MIT-BIH	1	ResNet18	×	×	68.81	83.39	86.60	84.83
	2	ResNet18	√	×	82.95	86.18	88.23	88.33
	3	ResNet18	×	√	85.67	86.51	84.70	85.08
	4	ResNet18	√	√	**91.03**	**91.22**	**91.90**	**92.07**
CPSC2018	1	ResNet18	×	×	31.85	31.80	72.57	71.92
	2	ResNet18	√	×	76.07	75.12	81.47	80.56
	3	ResNet18	×	√	50.28	51.16	81.44	81.22
	4	ResNet18	√	√	**83.01**	**82.64**	**85.34**	**85.07**

**Table 8:** Comparison of results of different adversarial training methods (%).

Dataset	Method	PGD		SAP	
		${ACC}_{robust}$	${F1}_{robust}$	${ACC}_{robust}$	${F1}_{robust}$
MIT-BIH	Adversarial Training	91.02	91.11	92.06	92.50
	Trades	88.04	88.18	89.34	89.49
	MART	**91.03**	**91.22**	**91.90**	**92.07**
CPSC2018	Adversarial Training	52.34	51.97	79.05	78.76
	Trades	74.58	73.82	83.23	82.91
	MART	**83.01**	**82.64**	**85.34**	**85.07**

**Table 9:** Contrast results with different methods in MIT-BIH dataset (%).

Method	PGD		SAP	
	${ACC}_{robust}$	${F1}_{robust}$	${ACC}_{robust}$	${F1}_{robust}$
VGG19	35.85	36.94	63.69	64.31
VGG19+MART	82.21	82.30	80.04	83.91
ResNet18	56.79	57.66	65.78	65.97
ResNet18+ MART	68.81	83.39	86.60	84.83
DenseNet	71.18	69.20	79.91	78.27
DenseNet + MART	83.51	83.61	85.31	85.39
CASLCNet	53.04	51.35	65.18	64.47
CASLCNet + MART	**91.03**	**91.22**	**91.90**	**92.07**

**Table 10:** Contrast results with different methods in CPSC2018 dataset (%).

Method	PGD		SAP	
	${ACC}_{robust}$	${F1}_{robust}$	${ACC}_{robust}$	${F1}_{robust}$
VGG19	21.95	23.19	66.14	65.19
VGG19+MART	30.17	30.23	70.10	67.26
ResNet18	18.42	18.43	39.47	38.76
ResNet18+ MART	31.85	31.80	72.57	71.92
DenseNet	22.22	21.89	74.00	73.48
DenseNet + MART	44.50	43.98	76.79	75.70
CASLCNet	46.76	45.99	84.76	84.74
CASLCNet + MART	**83.01**	**82.64**	**85.34**	**85.07**

**Table 11:** Comparative experimental results with other methods (%).

Author	Method	Dataset	PGD		SAP	
			${ACC}_{robust}$	${F1}_{robust}$	${ACC}_{robust}$	${F1}_{robust}$
Ma et al. [19]	Jabob	MIT-BIH	79.94	79.88	79.88	82.93
		CPSC2018	48.91	49.18	80.60	70.12
	SNR	MIT-BIH	88.64	88.63	88.63	91.08
		CPSC2018	46.99	46.67	81.26	80.98
Ours	CASLCNet	MIT-BIH	**91.03**	**91.22**	**91.90**	**92.07**
		CPSC2018	**83.01**	**82.64**	**85.34**	**85.07**

## Data Availability

The dataset used in this study is publicly available on Kaggle at https://www.kaggle.com/datasets/shayanfazeli/heartbeat and Icbeb at http://2018.icbeb.org/ (accessed on 30 April 2024).
